# Finite Element Analysis of Interfacial Debonding in Copper/Diamond Composites for Thermal Management Applications

**DOI:** 10.3390/ma10070739

**Published:** 2017-07-02

**Authors:** Muhammad Zain-ul-abdein, Hassan Ijaz, Waqas Saleem, Kabeer Raza, Abdullah Salmeen Bin Mahfouz, Tarek Mabrouki

**Affiliations:** 1 Mechanical Engineering Department, University of Jeddah, Jeddah 21589, Saudi Arabia; hassan605@yahoo.com (H.I.); waqas95@yahoo.com (W.S.); 2 FMSE, GIK Institute of Engineering Sciences and Technology, Topi 23640, Pakistan; hkabeerraza@gmail.com; 3 Chemical Engineering Department, University of Jeddah, Jeddah 21589, Saudi Arabia; asbinmahfouz@uj.edu.sa; 4 Mechanical Engineering Department, ENIT, University of Tunis El Manar, Tunis 2092, Tunisia; tarek.mabrouki@enit.rnu.tn

**Keywords:** finite element analysis, copper/diamond composite, interfacial debonding, thermal cyclic load, Cr-coated diamond

## Abstract

Copper/diamond (Cu/D) composites are famous in thermal management applications for their high thermal conductivity values. They, however, offer some interface related problems like high thermal boundary resistance and excessive debonding. This paper investigates interfacial debonding in Cu/D composites subjected to steady-state and transient thermal cyclic loading. A micro-scale finite element (FE) model was developed from a SEM image of the Cu/20 vol % D composite sample. Several test cases were assumed with respect to the direction of heat flow and the boundary interactions between Cu/uncoated diamonds and Cu/Cr-coated diamonds. It was observed that the debonding behavior varied as a result of the differences in the coefficients of thermal expansions (CTEs) among Cu, diamond, and Cr. Moreover, the separation of interfaces had a direct influence upon the equivalent stress state of the Cu-matrix, since diamond particles only deformed elastically. It was revealed through a fully coupled thermo-mechanical FE analysis that repeated heating and cooling cycles resulted in an extremely high stress state within the Cu-matrix along the diamond interface. Since these stresses lead to interfacial debonding, their computation through numerical means may help in determining the service life of heat sinks for a given application beforehand.

## 1. Introduction

Copper/diamond (Cu/D) composites have recently gained popularity in thermal management applications due to their improved thermal conductivity (TC) and high heat dissipation characteristics. They, however, suffer from an increased level of interfacial resistance and debonding, where the latter is a direct consequence of the difference between coefficients of thermal expansion (CTEs) of copper (~16.7 × 10^−6^ K^−1^) and diamond (~0.89 × 10^−6^ K^−1^). An adequate solution to this problem is the introduction of an interface medium with some intermediate value of CTE, for example chromium (~6.1 × 10^−6^ K^−1^). Such an interface material is likely not only to reduce the CTE mismatch, but also to improve the bond strength between the Cu-matrix and diamond particles. Given that the TCs of Cu and diamond are on the order of 400 W·K^−1^·m^−1^ and 2000 W·K^−1^·m^−1^, respectively, their combination (Cu/D) should theoretically yield the TC values such that TC_D_ > TC_Cu/D_ > TC_Cu_. Nevertheless, the interfacial boundary resistance plays its role and at times lowers the TC of the composite to the values less than that of Cu. This problem is taken care of by introducing some appropriate intermediate material between the matrix and the inclusion.

In an attempt to improve the interfacial bond strength and reduce the boundary resistance, several authors [1,2,3,4,5,6,7] exploited the so-called ‘pre-coated’ diamonds, where the coating material was chromium, tungsten, titanium, molybdenum, etc. It should be noted that the bond strength and, hence, the resulting TC of the composite is mainly a function of the coating material properties, fabrication method, thickness of coating, and the type of bond (physical or chemical). For example, Mizuuchi et al. [8], using Cu-coated diamond (CuD), achieved an increase in conductivity from 300 to 654 W·K^−1^·m^−1^ by changing the sintering temperature from 700 to 900 °C. Kang et al. [9] reported conductivity values approaching 562 W·K^−1^·m^−1^, where Cr was coated upon diamond through a molten salt method, while Ren et al. [2] used a vacuum deposition method for Cr coating and found a conductivity of 657 W·K^−1^·m^−1^. Zhang et al. [1] examined the effect of titanium coating and observed an improvement in TC from 170 to 493 W·K^−1^·m^−1^. Abyzov et al. [10], on the other hand, noticed a decrease in conductivity from 910 to 480 W·K^−1^·m^−1^ as a function of the increase in the tungsten coating thickness from 110 to 470 nm. For further description on TC of diamond composites and their fabrication by powder metallurgy, the readers are referred to the works of Kidalov et al. [11] and Hamid et al. [12].

Finite element (FE) analysis has proven itself to be a useful tool for capturing the behavior of materials, which is otherwise difficult to determine through experimental means. Bakker [13] used the FE method to calculate the thermal and electrical conductivities of composite materials. Decarlis and Jaeger [14] proposed a self-consistent FE model to compute the effective TC of a heterogeneous two phase medium. Yamada et al. [15] performed both the experimental and FE analyses to develop highly thermally conductive fiber-reinforced SiC composites. Similarly, Yang et al. [16] and Rape et al. [17] used experimental and numerical techniques to predict the effective TCs of Al/diamond and Cu/Zr-coated diamond composites, respectively. The capabilities of the FE method have been exploited in the present work to predict the interfacial debonding of the Cu/D composite at the micro-scale.

## 2. Scope and Methodology

Given that heat sinks allow rapid dissipation of heat energy from the microelectronic components, it is desirable that their conductive properties, when ‘in-service’, do not deteriorate as a result of thermal fatigue/cyclic loading. Since the interfacial debonding can lead to the degradation of material properties, the present work primarily deals with the FE analysis of a Cu/D composite subjected to thermal loading. The initial phases of this work were dedicated to, (i) the fabrication of Cu/D composites using uncoated, Cu-coated (CuD), and Cr-coated diamonds (CrD); (ii) the experimental identification of effective TC and optimization of sintering parameters [7]; and (iii) the numerical investigation of the effect of the interfacial boundary resistance upon the effective TC [18].

**Figure 1:**
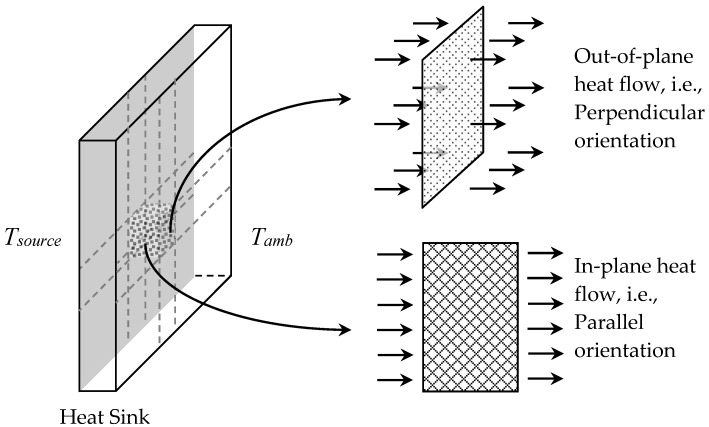
Schematic of the heat sink indicating the heat flow from source to ambient, such that *T_source_* > *T_amb_*, in the perpendicular and parallel orientations.

This paper describes the 2D micro-scale FE modeling approach in detail. A micro-scale thermo-mechanical analysis requires mesh generation from SEM images. Since SEM images provide only a 2D representation of the composite samples, the direction of heat flow with respect to the 2D micrograph can be anywhere between perpendicular (out-of-plane) or parallel (in-plane) orientation, see Figure 1. Hence, the cases of perpendicular (PD) and parallel (PL) orientations are the limiting cases.

Now, the PD orientation of a 2D FE model suggests a transient heat transfer analysis, where the temperature of the entire domain (SEM image) increases and decreases simultaneously, i.e., no in-plane thermal gradient ($\vec{\mathrm{grad}}\;T=0$). Conversely, the PL orientation, given a prolonged heating, will eventually lead to a steady-state condition, resulting in a strong thermal gradient ($\vec{\mathrm{grad}}\;T\neq 0$) depending upon the source and ambient temperatures.

Considering the interfacial resistance and debonding, the resulting expansion/contraction of the Cu-matrix, diamond-particles, and the interface material will give rise to several types of thermo-mechanical analyses, which are summarized as case studies in Table 1 in the order of increasing complexity.

## 3. Experimental Work

A detailed account of experimental work is presented in [7]. Only a brief description relevant to the current investigation is being provided here. Using uncoated and Cr-coated diamonds (CrD), Cu/D composites were fabricated by a powder metallurgy technique, convention sintering, in a vacuum tube furnace with diamond volume fractions between 5–20%. The particle size of cubo-octahedral shaped diamond and irregular copper powders were 100 μm and 45 μm, respectively. The Cr coating was performed by the diffusion method that produced a layer of thickness between 0.3 and 1 μm. Finally, the TC of each sample was measured using the principal of transient plane source method.

**Figure 2:**
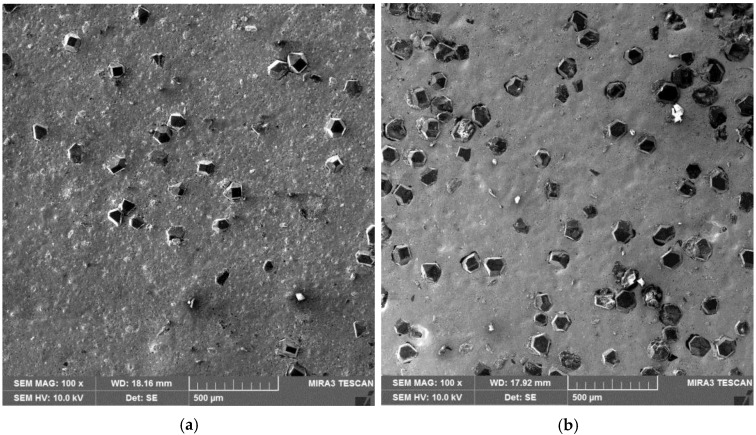
SEM images of the Cu/CrD composite with diamond fractions—(**a**) 5 vol %; (**b**) 20 vol %.

The SEM images of composite samples that can be used for numerical modeling are shown in Figure 2. By and large, the distribution of diamond particles in the Cu matrix is uniform throughout. Some distinctions, however, can be made with respect to the specific volume fraction. For example, Figure 2a with 5 vol % diamond shows large areas of continuous matrix, while Figure 2b with 20 vol % diamond illustrates frequent clusters of several diamond particles. From the viewpoint of the FE simulation, Figure 2b is of greater interest since it has a higher degree of matrix discontinuity and is likely to reveal maximum information regarding the debonding behavior. It is for this reason that the Cu/20 vol % diamond composite, corresponding to Figure 2b, was selected for FE analysis.

## 4. Finite Element Analysis

### 4.1. Thermo-Mechanical Model

#### 4.1.1. Coupling Phenomenon

FE analysis was carried out using the commercial software Abaqus/Standard (Abaqus, 6.16, Dassault Systemes, Johnston, RI, USA, 2016). In the case of thermo-mechanical analysis, Abaqus offers two types of coupling called sequential coupling and full coupling. The term ‘sequential’ refers to the calculation of the nodal temperature values through a heat transfer analysis at first. These temperature values are then fed to the stress analysis as a pre-defined field and the mechanical response is determined. Note that in a sequentially coupled analysis, the mechanical analysis cannot affect the temperature values, since the coupling exists from thermal to mechanical analysis and there is no reverse coupling. The sequentially coupled analysis is important where the plastic strain rate’s effect upon the change in temperature is trivial.

A fully coupled temperature-displacement analysis, however, estimates the thermal and mechanical responses simultaneously, such that the coupling exists from thermal to mechanical as well as from mechanical to thermal analyses. A fully coupled analysis is selected where the mechanical dissipation (i.e., specific plastic energy: $\int \sigma \cdot d\varepsilon^{p}$) due to very high strain rates is strong enough to cause a change in temperature. Another reason for choosing a fully coupled analysis is the ease of model development from the user’s point-of-view, since this analysis eliminates the need of making two separate models, one for each thermal and mechanical analysis. For this reason, fully coupled analyses were performed in each test case.

#### 4.1.2. Thermal Analysis

Computation of the temperature (*T*) field in a 2D (*x*, *y*) space and time (*t*) requires the solution of the following heat equation:

(1) $$\frac{\partial }{\partial x}\left(\lambda \left(T\right)\frac{\partial T}{\partial x}\right)+\frac{\partial }{\partial y}\left(\lambda \left(T\right)\frac{\partial T}{\partial y}\right)+Q_{\mathrm{int}}\;=\;\rho \left(T\right)C_{p}\left(T\right)\left(\frac{\partial T}{\partial t}\right)$$

where, λ, ρ, and $C_{p}$ are the thermal conductivity (W·K^−1^·m^−1^), density (kg·m^−3^), and specific heat (J·kg^−1^·K^−1^), respectively, all as functions of temperature. Also, $Q_{\mathrm{int}}$ is the internal heat generation rate in W·m^−3^. In the current FE model, the materials (Cu and diamond) were defined as separate parts. Hence, it was necessary to define the material properties of each constituent of the composite individually and not as the effective properties of the overall composite. Table 2 reports the temperature dependent density, thermal conductivity, and specific heat of the copper and diamond for the heat transfer analysis.

#### 4.1.3. Mechanical Analysis

The elastic-plastic analysis is required for the computation of the stress and strain states. In the present work, the diamond particles were assumed to be deformed elastically, while the Cu-matrix was assumed to follow elasto-plastic deformation with isotropic hardening behavior. For the case of thermo-elasto-plastic deformation, the total strain (ε) may be decomposed into elastic ($\varepsilon^{e}$), thermal ($\varepsilon^{t}$), and plastic ($\varepsilon^{p}$) parts as follows:

(2) $$\varepsilon =\varepsilon^{e}+\varepsilon^{t}+\varepsilon^{p}$$

The elastic part of the strain tensor can be defined with the help of Young’s modulus (*E*(*T*)) and Poisson’s ratio (*ν*).

The thermal strain can be expressed in terms of the reference temperature (*T*_0_) and the temperature dependent CTE (*α(T)*) as in the following:

(3) $$\varepsilon^{t}=\alpha (T)\left(T-T_{0}\right)$$

Note that the CTE (*α(T)*) in the above equation provides coupling between the thermal and mechanical analysis, such that any change in temperature will cause mechanical deformation. Hence, the solution of Equation (3) is a must for any type of coupled thermo-mechanical analysis.

In order to model the non-reversible deformation of the matrix, it is imperative to define the plasticity criterion. One of the most common criteria is known as the Mises plasticity criterion. Mises defined a yield function (*f*) to limit the elastic region, such that:

(4) $$f\left(\sigma ,T,H_{iso}\right)=\sigma -\sigma_{y}\;;\;\left\{ \begin{array}{l} f<0\;\Rightarrow \;Elastic\;deformation\\ f=0\;\Rightarrow \;Yielding \end{array}\right.$$

where, $H_{iso}$ is the isotropic hardening parameter. Equation (4) implies that when the given stress is equal to the yield stress, *f* = 0. Any further increase in the stress value results in an expansion of the yield surface because of the isotropic hardening behavior. The plastic flow of the material is defined using the flow rule:

(5) $${\dot{\varepsilon }}^{p}=\frac{3}{2}\dot{p}\frac{S}{\sigma_{eff}}\;\text{with}\;\sigma_{eff}=\sqrt{\frac{3}{2}S:S}=\sqrt{\frac{1}{2}\left[{\left(\sigma_{1}-\sigma_{2}\right)}^{2}+{\left(\sigma_{2}-\sigma_{3}\right)}^{2}+{\left(\sigma_{3}-\sigma_{1}\right)}^{2}\right]}$$

where, ${\dot{\varepsilon }}^{p}$ is the plastic strain rate, $\sigma_{eff}$ is the von Mises effective stress, *S* is the deviatoric stress, and $\dot{p}$ is the flow rate that adjusts itself at a given plastic strain rate to satisfy the consistency condition *f* = 0. $\sigma_{1}$, $\sigma_{2}$, and $\sigma_{3}$ are the three principal stresses.

#### 4.1.4. Yield Strength

Although the yield strength ($\sigma_{y}$) dependency upon the strain rate is less significant in thermal management applications, especially for low heating and cooling rates, if however the effect is included, the yield stress may be expressed as $\sigma_{y}=\sigma_{y}\left(\varepsilon^{p},{\dot{\varepsilon }}^{p},T\right)$. As mentioned earlier, the Cu-matrix deforms in an elastic-plastic regime, and its yield strength evolution may be written as follows [25]:

(6) $$\sigma_{y}(\varepsilon_{p},{\dot{\varepsilon }}_{p},T)=\underset{Plastic\;term}{\underset{︸}{\left(90+292\varepsilon^{p^{0.31}}\right)}}\times \underset{Rate-dependent\;term}{\underset{︸}{\left(1+0.025\ln\left({\dot{\varepsilon }}^{p}\right)\right)}}\times \underset{Temperature-dependent\;term}{\underset{︸}{\left(1-{\left(\frac{T-T_{0}}{1083-T_{0}}\right)}^{1.09}\right)}}$$

Equation (6) illustrates the dependency of the yield strength upon three significant factors, namely the plastic strain ($\varepsilon^{p}$), strain-rate (${\dot{\varepsilon }}^{p}$), and temperature (T). Here, the rate-dependent term is directly proportional to the yield strength, hence the increase in the plastic deformation rate will strain harden the Cu-matrix. On the other hand, the temperature-dependent term is in inverse proportion to that of $\sigma_{y}$, which implies that the increase in temperature will lead to the softening of the matrix. Note that at $T=T_{0}$ and ${\dot{\varepsilon }}^{p}=1$, $\sigma_{y}$ will follow a power law form for the evolution of the hardening curve as a function of $\varepsilon^{p}$ only. This means that at a low operating temperature and strain rate, it is safe to assume that $\sigma_{y}=\sigma_{y}\left(\varepsilon^{p}\right)$. Nevertheless, in the present work the complete form of Equation (6) has been used.

The mechanical properties for the Cu-matrix and the diamond reinforcement are provided in Table 2.

### 4.2. Microstructure Based Modelling

**Figure 3:**
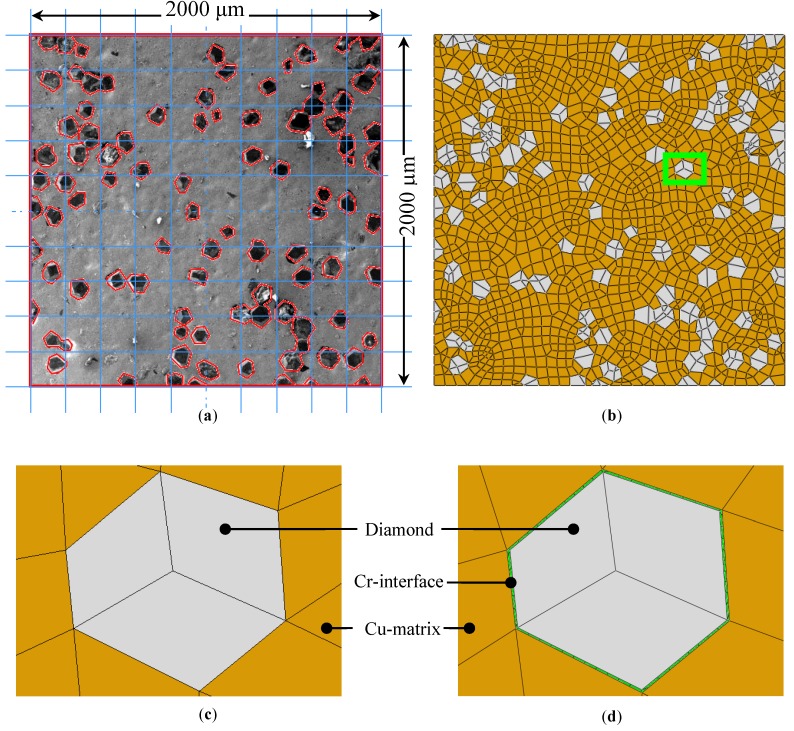
Mesh generation—(**a**) SEM image of the Cu/20 vol % CrD composite; (**b**) Finite element (FE) mesh; (**c**) Cu/D composite mesh without the Cr-interface; and (**d**) Cu/D composite mesh with the Cr-interface.

The thermo-mechanical analysis necessitates the use of elements with temperature and displacement degrees of freedom. A 2D model, with the overall dimensions of 2000 μm × 2000 μm (Figure 3a), was developed using SEM images of the Cu/20 vol % diamond composite. Figure 3b shows the micro-scale mesh generation that is comprised of more than 2200 nodes and 1700 elements. The elements of type CPE4RT (4-node bilinear coupled temperature-displacement plane strain quadrilateral element with reduced integration and hourglass control) and CPE3T (3-node linear coupled temperature-displacement plane strain triangular element) were used throughout. Abaqus/Standard offers reduced integration in quadrilateral elements with only one integration point at the centroid. These elements are, therefore, also referred to as centroid strain elements.

Two types of meshes were used for the different cases. Type-I mesh, corresponding to the cases 1, 2, and 4 (Table 1), was defined for the Cu/D composite without the interface layer, whereas type-II mesh, corresponding to case 3 (Table 1), was generated for the Cu/CrD composite with the interface layer. Figure 3c,d, referring to the types I and II meshes, respectively, illustrate the magnified view of the region highlighted in Figure 3b. It may be noticed that the interface layer thickness differs by a factor of 100 with respect to the average size of the diamond particles. This is consistent with the previously mentioned experimental observation.

### 4.3. Interactions and Boundary Conditions

Micro-modeling of a two phase heterogeneous composite requires treatment of both the constituents, i.e., matrix and filler, as interacting media. In Abaqus, the interactions may either be defined as *TIE constraint or *CONTACT pair properties. Here, the former is a case of perfect contact, while the latter can accommodate interfacial properties like cohesion, friction, etc. Note that the interfacial resistance and the bonding/debonding can be defined in terms of the thermal conductance and the cohesive behavior, respectively. Precisely in the context of thermo-mechanical analysis, both the thermal and mechanical contact properties must be defined. Hence, the thermal conductance will be regarded as a part of the thermal contact modeling, while the cohesive interaction will be treated as the mechanical contact property.

In case of absolute conductance and perfect bonding between the matrix and inclusions, the complete domain can be modeled as a single ‘part’ with different material properties in the prescribed regions and no internal surface at all. This situation refers to cases 1 and 4 in Table 1. However, if a non-zero interfacial resistance and the debonding behavior are to be included, the composite must be modeled in two parts, i.e., matrix and inclusions, so as to generate the internal surfaces and, hence, the interactions. Case 2 takes both the interactions into account.

A relatively complex situation arises if a third part, the interface layer, is to be modeled (case 3). One of the functions of the interface layer is to improve the adhesion between the matrix and reinforcements. Consequently, it may be defined as a cohesive material with some appropriate damage model. In the present case, however, the Cr reacts chemically with diamond to form a strongly adherent compound of type Cr_*x*_C_*y*_ [7], which is highly unlikely to damage/chip-off as a result of the heating and cooling of the heat sinks. On the contrary, the debonding of Cr with the Cu-matrix can still be expected provided that the thermal stresses are significantly high. The tie constraint must, therefore, be defined between diamond and Cr, whereas the cohesive behavior should be incorporated between Cu and diamond.

**Figure 4:**
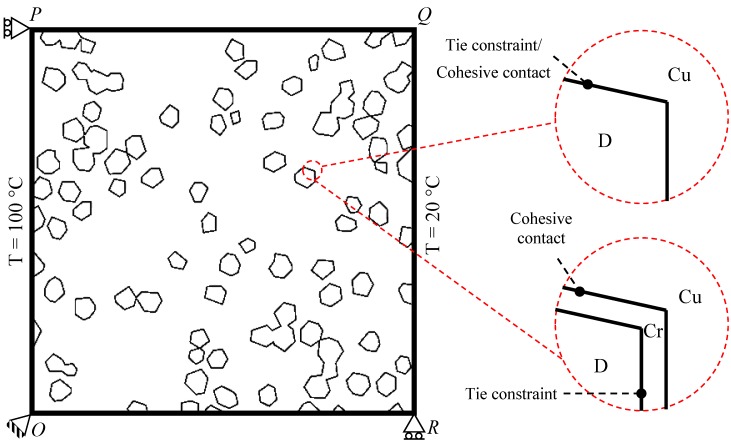
Schematics of the boundary conditions and the types of interfaces—Tie constraint or the cohesive contact at the Cu/uncoated diamond interface, tie constraint at the Cr/diamond interface, and cohesive contact at the Cu/Cr interface.

The boundary conditions (BCs) and the interactions used in different test cases are shown in Figure 4 and are explained in the following:

• Case 1 - Thermal BCs: Prescribed temperatures on the edges *OP*: 100 °C; *QR*: 20 °C
  - Mechanical BCs: Displacements at vertices *O*: U*x* = U*y* = 0; *P*: U*x* = 0; *R*: U*y* = 0
  - Interactions: Perfect contact, absolute conductance
• Case 2 - Thermal BCs: Prescribed temperatures on the edges *OP*: 100 °C; *QR*: 20 °C
  - Mechanical BCs: Displacements at vertices *O*: U*x* = U*y* = 0; *P*: U*x* = 0; *R*: U*y* = 0
  - Interactions: COHESIVE contact between D and Cu
• Case 3 - Thermal BCs: Prescribed temperatures on the edges *OP*: 100 °C; *QR*: 20 °C
  - Mechanical BCs: Displacements at vertices *O*: U*x* = U*y* = 0; *P*: U*x* = 0; *R*: U*y* = 0
  - Interactions: TIE constraint at Cr/D interface, COHESIVE contact between Cr and Cu
• Case 4 - Thermal BCs: Cyclic BC on all nodes, Max. T: 160 °C; Temperature rate: 100 °C/s
  - Mechanical BCs: Displacements at vertices *O*: U*x* = U*y* = 0; *P*: U*x* = 0; *R*: U*y* = 0
  - Interactions: Perfect contact, absolute conductance

It is important to note that the prescribed temperature was selected from 100 °C for steady-state analysis up to 160 °C for transient analysis. This is because the working temperature in typical electronic components ranges from 50 to 300 °C [26]. Similarly, in automobiles, the operating temperature of logic devices is less than 200 °C [27]. Moreover, in military applications, some electronic devices need to maintain a working temperature of less than 125 °C for ideal operation [28].

### 4.4. Cohesion/Separation Criterion

In order to quantify the effect of the interfacial Cr layer, it is essential to define the cohesive interface in terms of the normal and shear penalty stiffnesses, *K_i_*, *i* = 1, 2, or 3. Here, the penalty stiffness, *K_i_*, is defined as a ratio between the corresponding normal or shear interfacial strength, *σ_i_*, and the critical separation for damage initiation, *D_i_*. For the composite laminates, Turon et al. [29] proposed that the penalty stiffness may be estimated by using the relation $K_{i}\geq \alpha_{T}E_{i}/\delta$, where *E_i_* are the elastic constants, *δ* is the thickness of the sub-laminate, and *α_T_* is the Turon parameter whose value depends upon the requirement to avoid interpenetration of the separating surfaces. Note that in the present work, the Cr layer was not modeled as a laminate, but rather a surface based cohesive behavior was defined at the interface of the Cu-matrix and Cr layer (see Figure 4) using a built-in module of Abaqus. The surface based cohesive behavior assumes a linear elastic traction-separation law with damage initiation and evolution criteria, where the elastic properties (*K_i_*) depend, by default, upon the underlying element stiffness. The damage initiation and evolution were based upon the maximum stress criterion and the separation of interacting surfaces, respectively.

## 5. Results and Discussion

### 5.1. Steady-State Analysis without Interacting Surfaces—Case 1

The main objective here is to evaluate the equivalent Mises stress and plastic strain induced by heating the Cu/diamond composite in the PL orientation such that $\vec{\mathrm{grad}}\;T\neq 0$.

#### 5.1.1. Temperature Contours

**Figure 5:**
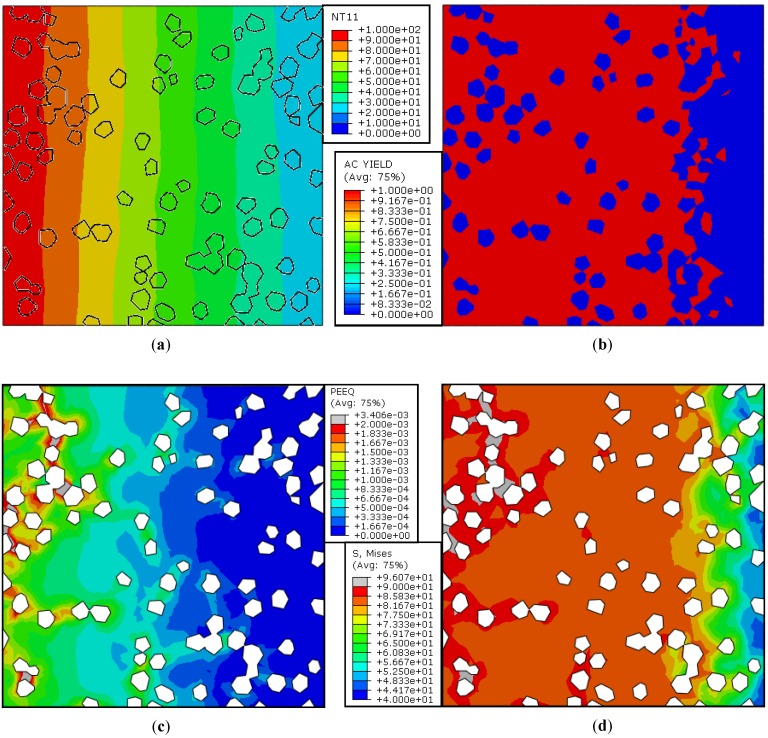
Steady-state analysis without interacting surfaces (Case 1): Contours of (**a**) Nodal temperature (°C); (**b**) Active yielding; (**c**) Equivalent plastic strain; (**d**) Mises equivalent stress (MPa).

Figure 5a illustrates the nodal temperature contours over the entire domain. Given the prescribed thermal BCs (Figure 4), the contours lines should remain parallel to the vertical axis in the absence of any second phase inhomogeneities. Although in the present case the second phase particles, i.e., diamonds, offer no boundary resistance, they differ from the Cu-matrix with respect to the thermal and mechanical properties (see Table 2). Note that the non-parallel temperature contour lines are purely due to the difference in material properties of the matrix and inclusions.

#### 5.1.2. Actively Yielding Regions

Diamond is known to be the hardest material in nature. It is, therefore, obvious that only the Cu-matrix will undergo plastic deformation as a result of the heating and cooling cycle. Figure 5b shows the regions of active yielding within the mesh. It may be noticed that the diamond particles are unyielding throughout, or in other words, they experience elastic deformation only. In the case of the Cu-matrix, however, the regions where the temperature goes beyond 40 °C are actively yielding, whereas the low temperature zone (*T* < 40 °C) deforms elastically. This means that the equivalent plastic strain should be present within the high temperature zone of the Cu-matrix only.

#### 5.1.3. Plastic Strain Distribution

Figure 5c presents the contours of equivalent plastic strain within the Cu-matrix. It is understood that the higher the temperature, the higher the plastic deformation. The presence of diamond particles in the high temperature zone, however, reveals that the regions of the matrix in the immediate vicinity of an unyielding impurity deforms relatively more than the rest of the ‘bulk’ matrix. This is because the diamond particles, first of all, behave in a manner similar to a rigid body; secondly, they are in a perfect contact with the Cu-matrix. In effect, when the composite is exposed to a significantly higher temperature, the elastically deforming diamond with a very small CTE (~0.89 × 10^−6^ K^−1^) does not allow for the expansion or separation of Cu having a high CTE (~16.7 × 10^−6^ K^−1^) and, as a result, Cu deforms with a relatively higher level of plastic strain next to the diamond particles.

#### 5.1.4. Mises Equivalent Stress Distribution

The resulting equivalent stress state in terms of Mises stress is shown in Figure 5d. Note that the peak stresses appear at the same locations where the plastic strain is maximum, i.e., next to the diamond particles within the high temperature zone. Note that from Equation (6) that $\sigma_{y}$ = 90 MPa at $\varepsilon^{p}$ = 0. Figure 5d shows that the maximum stress level is more than 90 MPa within the high temperature regions entrapped between the clusters of diamond particles. Being at the Cu/diamond interface, the highly stressed plastically deforming regions are, in fact, the potential debonding sites within the composite. It should also be pointed out here that no matter how perfect the bonding between the Cu and diamond is, the stress state of the Cu-matrix is high enough to cause permanent deformation and the major reason is the significant difference in expansion and contraction of both the constituents due to their different CTEs. This concludes that the stress distribution may well be influenced by choosing an appropriate interfacial medium that can result in the decrease in the difference of the CTEs of the materials in contact.

### 5.2. Steady-State Analysis with Interacting Surfaces—Case 2 vs. Case 3

**Figure 6:**
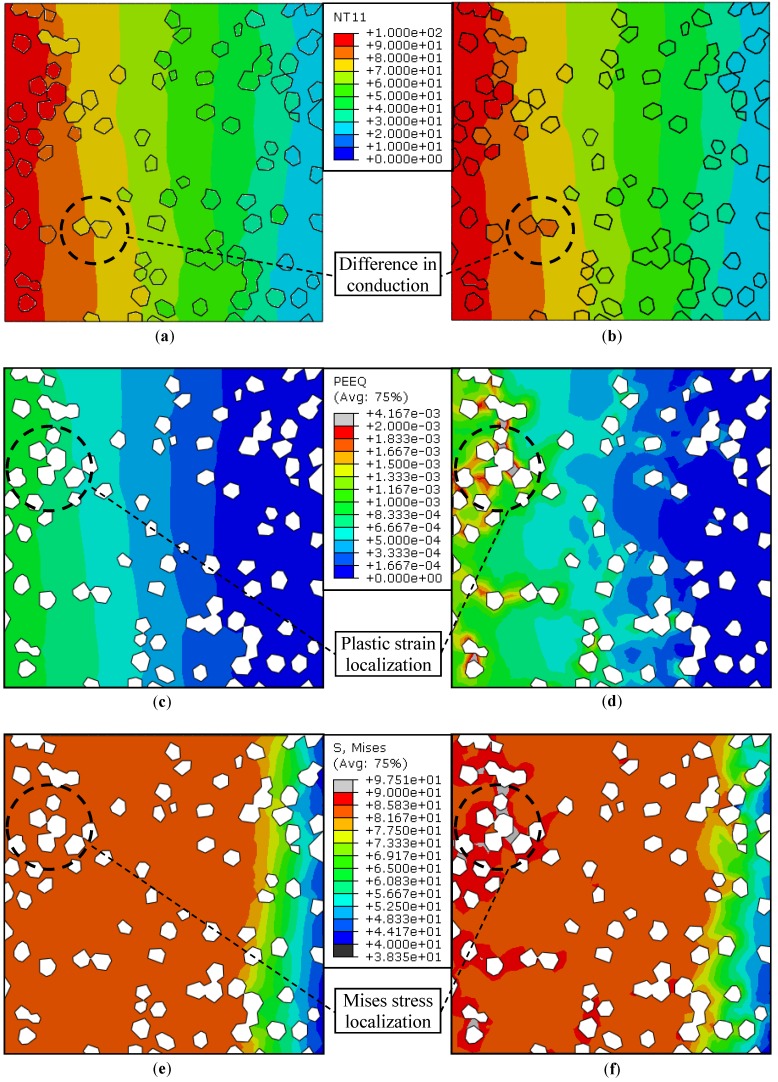
Steady-state analysis with interacting surfaces—Contours of (**a**) Case 2: Nodal temperature (°C); (**b**) Case 3: Nodal temperature (°C); (**c**) Case 2: Equivalent plastic strain; (**d**) Case 3: Equivalent plastic strain; (**e**) Case 2: Mises equivalent stress (MPa); and (**f**) Case 3: Mises equivalent stress (MPa).

The results of cases 2 and 3 are compared in Figure 6. It is important to note that in both cases, the matrix and the inclusions were modeled as separate entities with the interacting surfaces, whereas the only difference was the addition of the Cr interface layer in case 3. The details of the interactions have already been presented in Section 4.3; their effect upon the temperature, strain, and stress fields is discussed here.

#### 5.2.1. Temperature Contours

Figure 6a,b show the temperature contours in the steady-state condition for cases 2 and 3, respectively. Compared to case 1 (Figure 5a), cases 2 and 3 demonstrate relatively more irregular temperature profiles for two additional interface related reasons: the thermal resistance and the debonding. Note that a finite value of thermal conductance, 14 kW·m^−2^·K^−1^ [18], was used throughout, which resulted in a difference in temperatures across the Cu/diamond interface. Coupled with the difference in CTEs, an uneven expansion of the matrix and the reinforcement led to the interfacial debonding in the absence of ‘cohesion’ between the surfaces. Unlike the case of an ideal contact (case 1), case 2 is representative of a non-ideal contact with minimum bond strength, say <1 MPa, due to poor wettability of the diamond by Cu. The Cr, however, serves to improve the wetting characteristics of the interface between the Cu and diamond (case 3). For these reasons, though uneven, an improved heat transfer across the interface was observed in the Cu/CrD composite, which is indeed a desirable scenario for thermal management applications.

#### 5.2.2. Plastic Strain Distribution

The variations in equivalent plastic strains for both the cases are shown in Figure 6c,d. It may be observed that the peak strain in case 2 (Figure 6c) is less than that in case 1 (Figure 5c). This is because the cohesive properties in the former are almost insignificant as compared to the perfect contact in the latter case. Owing to a considerable difference in the CTEs of Cu and diamond, an interface that does not allow the debonding (case 1) will eventually deform the Cu-matrix. Conversely, an interface which can lead to the separation of the matrix and the inclusions, will limit the plastic deformation to a significantly lower level. This further suggests that in case 3 the interfacial Cr layer, with improved bonding characteristics and an intermediate CTE, is likely to yield a lesser degree of debonding as compared to case 2 at the cost of a higher level of equivalent plastic strain. Note that the difference in plastic strain levels is more pronounced in the high temperature zone (Figure 6c,d).

#### 5.2.3. Mises Equivalent Stress Distribution

The Mises equivalent stresses are more trivial in case 2 (Figure 6e) than in case 3 (Figure 6f), especially where the strain localization is important. It may be observed that the equivalent stress level does not exceed 90 MPa in case 2, while it surpasses this limit in the regions of high plastic strain. Recall that $\sigma_{y}$ = 90 MPa at $\varepsilon^{p}$ = 0. This means that although the Cr coating offers advantages like improved bonding, it may induce active yielding within the Cu/CrD composite (case 3) as compared to the Cu/D composite (case 2), under identical loading conditions. The stress localization that is induced is undesirable from the perspective of product life, and hence requires special care at the design stage.

#### 5.2.4. Interfacial Cohesion/Separation

**Figure 7:**
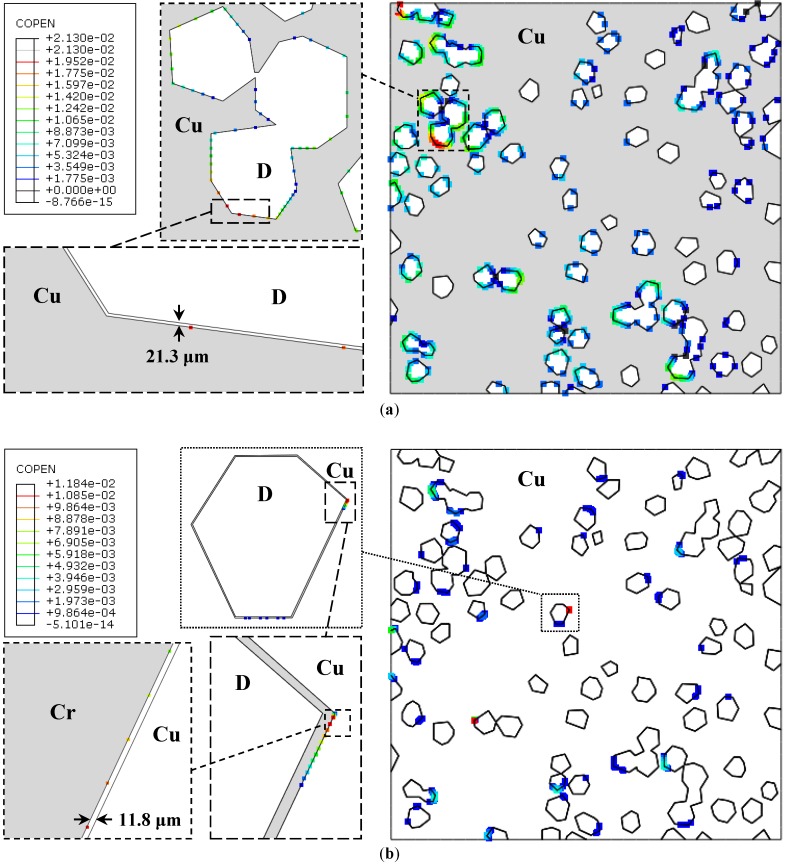
Contours of contact opening (mm)—(**a**) Case 2: Cu/uncoated diamond; (**b**) Case 3: Cu/Cr-coated diamond.

Figure 7a,b illustrate the successively enlarged debonding regions for cases 2 and 3, respectively, as a field variable ‘COPEN’, i.e., cohesive open. It may be observed that the maximum separation between the surfaces at the Cu/Cr-coated diamond interface is almost half (11.8 μm) of that of the Cu/uncoated diamond interface (21.3 μm). Moreover, the debonding is more pronounced in the high temperature zone. Also, it is more frequent in case 2 (Figure 7a) than in case 3 (Figure 7b). This is important because the only difference between the two composites is that of the interfacial coating material. Therefore, it may be stated that the debonding behavior depends not only upon the difference in CTEs but also upon the penalty stiffness/ elastic constants of the interface material.

### 5.3. Transient Analysis without Interacting Surfaces—Case 4

#### 5.3.1. Steady-State vs. Transient Analysis

Unlike the steady-state, a transient heat transfer analysis (∂T/∂t≠0) requires the definition of the specific heat capacity and density in addition to that of the thermal conductivity. Applied to the 2D model in the PD orientation (see Figure 1), the entire composite domain will experience the change in temperature simultaneously, i.e., $\vec{\mathrm{grad}}\;T=0$, during a complete heating and cooling cycle. Hence, for the transient analysis, both the maximum temperature ($T_{\max}$) as well as the temperature rate ($\dot{T}$) must be defined as BCs. Since the heat sinks go through several heating and cooling cycles throughout their service life, their failure due to thermal fatigue could be of interest for researchers. For instance, Figiel et al. [30] proposed a computational model for the composite delamination subjected to mechanical and thermal fatigue and predicted fatigue failure through FE analysis. Although the prediction of fatigue failure requires a fully dedicated investigation where cyclic properties of the composite, different temperature rates, number of cycles, maximum and minimum temperature, etc. may be included, a concise description is provided here to examine the effect upon the overall stress field. It should also be mentioned that in the present case the Cu-matrix and diamond particles were assumed to be in a perfect contact with each other similar to that in case 1, see Table 1.

#### 5.3.2. Yielding and Cyclic Response

**Figure 8:**
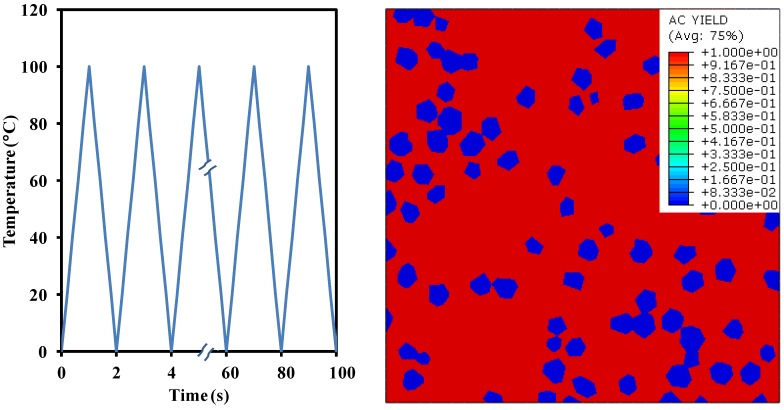
Case 4: Thermal boundary condition applied to all nodes (left), Contours of active yielding (right).

**Figure 9:**
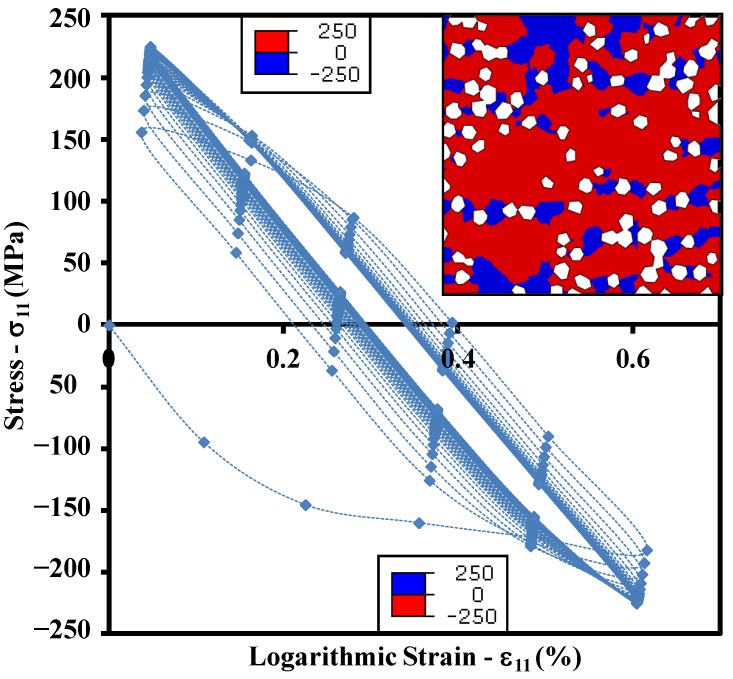
Case 4: Peak stress vs. logarithmic strain response to the applied thermal cyclic loading and zones of tension and compression within the Cu-matrix.

Figure 8 shows the cyclic thermal BC applied to all the nodes of the model. Note that the $T_{\max}=100{}^{\circ}C$, $\dot{T}=100{}^{\circ}C/s$, and the total number of cycles is 50. Figure 8 also illustrates the contours of active yielding at the end of the analysis, where only the Cu-matrix yielded fully while the diamond particles did not. The resultant cyclic maximum true stress-logarithmic strain curve calculated over the entire Cu-matrix in one of the normal directions is shown in Figure 9.

Given a fully random distribution of diamond particles and the symmetric BCs along both the normal axes, the change in cyclic behavior is negligibly small. It should be noted that during the first heating and cooling cycle, the Cu-matrix goes through compression and tension, respectively. This is because upon heating, the matrix with high CTE (~16.7 × 10^−6^ K^−1^) tries to expand in all directions, however its expansion is largely restricted by the rigid reinforcements of low CTE (~0.89 × 10^−6^ K^−1^), thereby inducing the compression in most elements. On the other hand, the cooling induces contraction of all the elements, yet the matrix shrinks more than the reinforcement due to the difference in CTEs. Once again, the rigid diamond particles do not allow the contraction of the Cu-matrix and hence induce tension in most of its regions. The tension and compression zones within the composite domain are also shown in Figure 9, such that the peak stress magnitude (250 MPa) does not change in either case. Note that the two different legends, one in compression and another one in tension, are shown for the same contour. This means that the regions which undergo compression during heating experience tension during cooling and vice versa, i.e., a complete reversal of the stress state is observed within each thermal cycle. It may be observed that the curve depicts cyclic hardening behavior that stabilizes within a few loading cycles. 

#### 5.3.3. Mises Equivalent Stress Distribution

**Figure 10:**
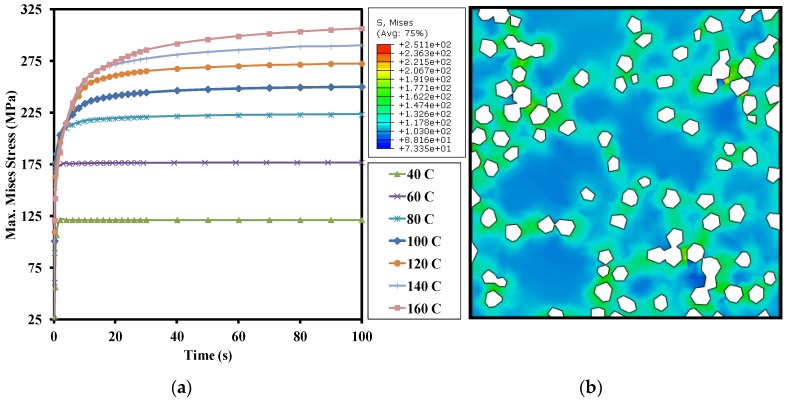
Case 4: Maximum von Mises equivalent stress as a function of time for different temperature values (**a**); Contours of Mises stress at the end of cyclic loading for a peak temperature of 100 °C (**b**).

In order to observe the evolution of the equivalent stress state, the maximum von Mises stress is plotted as a function of time in Figure 10 for different loading temperatures such that the peak temperature varies between 40 °C and 160 °C with increments of 20 °C. It should be mentioned that each curve refers to a separate simulation run, while each point on the curve corresponds to the maximum Mises stress within a complete thermal cycle.

It may be noted that the maximum equivalent stress increases from 125 MPa to 300 MPa with the increase in temperature from 40 °C to 160 °C, respectively. However, the peak stress value is present only in a few elements, whereas most of the elements experience significantly smaller stress levels. For instance, consider the Mises stress contours obtained at 100 °C, shown in Figure 10. The peak stress of almost 250 MPa is found at the location where the matrix is entrapped between the two clusters of diamond lying very close to each other. Excluding this region of peak stress, the maximum stress remains around 170 MPa at the Cu/diamond interface. Even this value of 170 MPa is almost twice as high as the maximum stress (~96 MPa) in case 1, see Figure 5d. The reasons for such a large variation in equivalent stress include the rate induced strain hardening as well as cyclic hardening in the present case. This implies that the thermal fatigue is more likely to lead to the debonding of the Cu/diamond interface as compared to the steady state thermal loading.

### 5.4. Comparison with Published Works

Although the published literature provides sufficient data for the TC values for various combinations of Cu/D composites [11], the information regarding the bond strength and its effect upon the serviceability of heat sink components is still rare to find. For instance, Hamid et al. [12] measured the transverse rupture strength (TRS) of the overall composite and observed that it varies from 210 to 270 MPa for uncoated diamonds, and from 439 to 653 MPa for NiWB-coated diamonds both as a function of the diamond volume fraction. Here, the TRS represents the fracture strength of the bulk material as well as the interfacial bond strength. In addition, the change in TRS values from uncoated to NiWB-coated diamonds reflects the presence of significant bond strength. Likewise, Kang et al. [9] showed an increase in bending strength as a result of Cr-coating from 100 MPa to 250 MPa.

Nevertheless, the question remains whether the CTE mismatch can generate enough thermal stresses that would lead to the interfacial debonding. In the present work, a numerical approach is presented to estimate the stress and strain states of the Cu/D composites subjected to steady-state and transient conditions that would eventually lead to the interfacial debonding, which is otherwise difficult to quantify through experimental means. This approach may be extended to several other combinations of materials in various proportions and under different thermal or mechanical BCs.

## 6. Conclusions

This work has been devoted to developing the micro-scale FE model for the prediction of interfacial debonding within the Cu/diamond composites subjected to thermal steady-state and transient cyclic loading. A total of four test cases with increasing complexity have been investigated. It was noted that although the source of interfacial debonding is the intrinsic CTE of each constituent of the composite, it also depends upon the bond strength between the matrix and the reinforcement. A perfect interfacial bond (case 1) leads to better heat conduction; however, it induces very high thermal stresses in its immediate vicinity. A weak interface (case 2) on the other hand yields poor conductive properties as well as a lower level of stresses in the matrix.

The numerical simulation can also be exploited to select the interfacial medium with intermediate CTE so as to predict the debonding behavior of the matrix and reinforcement. Applied to the Cr-coated diamonds immersed in the Cu-matrix (case 3), the interface separation (~11.8 μm) was found to be almost half (~21.3 μm) of that of the Cu/uncoated-diamond composite. This shows that an appropriate interface medium may further reduce the interfacial debonding and, hence, can help in improving the conductive properties of the composite. It was also revealed that the cyclic loading leads to a relatively higher level of thermal stresses within the matrix (case 4), however this depends strongly upon the heating and cooling rates. Nevertheless, a higher non-uniform state of stress will eventually lead to the interfacial debonding, thereby, degrading the overall properties of the composite.

From the perspective of the design and service life of heat sinks, this work may further be extended by integrating different interface material, thermal boundary conditions, composition, etc., with the numerical model. In addition, thermal fatigue failure may also be investigated by incorporating the material properties data corresponding to the cyclic behavior. Similarly, the effect of grain size/boundaries may also be included in future work.

## Figures and Tables

**Table 1:** Types of thermo-mechanical analyses.

Description	Case 1	Case 2	Case 3	Case 4
Composite	Cu/D ^1^	Cu/D	Cu/CrD ^1^	Cu/D
Analysis type	SS ^1^	SS	SS	TS ^1^
Model orientation	PL ^1^	PL	PL	PD ^1^
Interfacial resistance	NO	YES	YES	NO
Interfacial debonding	NO	YES	YES	NO

^1^ D: uncoated diamond; CrD: Cr-coated diamond; TS: transient; SS: steady-state; PD: perpendicular; PL: parallel.

**Table 2:** Material Properties of the Cu-matrix and diamond reinforcement.

Material Property (Units)	Copper		Diamond	
	Value	Ref	Value	Ref
Density (kg·m^−3^)	ρ(T)=8962.77−0.53T	[19]	$\begin{array}{l} \rho \left(T\right)=3516.2-0.019T\\ \;-2\times 10^{-5}T^{2} \end{array}$	[20]
Thermal conductivity (W·m^−1^·K^−1^)	$\begin{array}{l} \lambda \left(T\right)=389.45-3.05\times 10^{-5}T\\ \;-2.57\times 10^{-5}T^{2} \end{array}$	[19]	λ(T)=1477.6−4.581T	[21]
Specific heat (J·kg^−1^·K^−1^)	$C_{p}\left(T\right)=379.9+0.1T$	[22]	$\begin{array}{l} C_{p}\left(T\right)=532.47+2.67T\\ \;-1.1\times 10^{-3}T^{2} \end{array}$	[23]
CTE (K^−1^)	$\begin{array}{l} \alpha \left(T\right)=16.7\times 10^{-6}\\ \;+3.74\times 10^{-9}T \end{array}$	[19]	$\begin{array}{l} \alpha \left(T\right)=-1.24\times 10^{-12}T^{2}\\ +3.87\times 10^{-9}T+0.89\times 10^{-6} \end{array}$	[20]
Young’s Modulus (GPa)	E(T)=129.13−0.0668T	[19]	E=1220	[24]
Poisson’s ratio	ν=0.34	[19]	ν=0.2	[24]
Yield strength (MPa)	Equation (6)	[25]	$\sigma_{y}=1200$	[24]
